# Educational Case: Neuromyelitis optica

**DOI:** 10.1016/j.acpath.2022.100041

**Published:** 2022-08-16

**Authors:** Nathaniel Kitchens, Larry Nichols, Thomas Hope

**Affiliations:** Mercer University School of Medicine, Macon, GA, USA

**Keywords:** Pathology competencies, Diagnostic medicine, Immunologic mechanisms, Autoimmune diseases, Neuromyelitis optica spectrum disorder, Multiple sclerosis, Aquaporin 4, Apheresis

The following fictional case is intended as a learning tool within the Pathology Competencies for Medical Education (PCME), a set of national standards for teaching pathology. These are divided into three basic competencies: Disease Mechanisms and Processes, Organ System Pathology, and Diagnostic Medicine and Therapeutic Pathology. For additional information, and a full list of learning objectives for all three competencies, see https://www.journals.elsevier.com/academic-pathology/news/pathology-competencies-for-medical-education-pcme.^1^

## Primary objective

Objective IMM1.4: Autoimmune Diseases. Discuss the clinical presentation and pathophysiologic bases of autoimmune diseases including the efficient use of laboratory tests to make a definitive diagnosis and manage the disease.

Competency 3: Diagnostic Medicine and Therapeutic Pathology; Topic: Immunology (IMM); Learning Goal 1: Pathogenesis, Diagnosis, and Treatment of Immunologic Disorders.

## Secondary objective

Objective TM1.5: Apheresis. Explain the clinical role of therapeutic apheresis in the management of the following disorders: sickle cell anemia, thrombotic thrombocytopenia, acute and chronic inflammatory demyelinating polyneuropathy, myasthenia gravis, antiglomerular basement membrane disease, organ transplantation, plasma cell dyscrasias, leukemia, and lymphoma.

Competency 3: Diagnostic Medicine and Therapeutic Pathology; Topic: Transfusion Medicine (TM); Learning Goal 1: Concepts of Blood Transfusion.

## Patient presentation

A 25-year-old woman presents with a 7-day history of slowly progressive weakness in both lower extremities, numbness in both legs, and urinary urgency with incontinence. The patient noted that the weakness was primarily in the proximal muscles of the lower extremities and not ascending. She became unable to walk 5 days prior and had to be carried into the examination room by her boyfriend. She has had no back pain, neck pain, recent falls or injury.

The patient had a past medical history of headaches associated with blurred vision in one eye or the other for over a year. The visual disturbance occasionally lasted for several days. The headaches were most often unilateral, moderate to severe, and throbbing, with associated nausea and photophobia. Magnetic resonance imaging (MRI) of the brain without gadolinium enhancement performed one year prior to her current presentation was normal. The patient was prescribed rizatriptan as needed for headache, which she used 10–12 times over the course of the year. The patient is of Asian descent and does not use alcohol, tobacco, or illicit drugs.

## Diagnostic findings, Part 1

On examination, her temperature is 37 °C, pulse 76 beats/min, blood pressure 110/70 mm Hg, and respirations 14 breaths/min. Mental status and cranial nerve examinations are normal. Upper extremity sensory and upper motor examination are normal. She has severe right lower extremity weakness of all muscle groups (2/5) and moderate left lower extremity weakness (3/5). The patient can move her right leg but cannot overcome gravity. She is unable to walk. She has loss of vibratory and position sensation in both lower extremities and bilateral loss of pain and temperature sensation beginning at the T1 dermatome that is more prominent on the left. Upper extremity distal tendon reflexes (DTRs) are normal and lower extremity DTRs are hyperactive with bilateral positive Babinski responses.

## Questions/discussion points, Part 1

### Based on the history of the present illness and physical examination, what is the most likely diagnosis (which may be a syndrome or category of disease rather than a specific diagnosis)?

Limited to the current presentation, the patient has a combination of motor deficits, sensory deficits, and bladder symptoms. She does not have slurred speech, altered mental status, or deficits limited to one side of the body, and the onset is not sudden, so stroke is unlikely. The fact the patient has a T1 sensory level and that pain and temperature sensory loss is more prominent in the less weak leg localizes the lesion to the spinal cord. The patient's symptoms had a subacute onset over the course of a week, so a tumor or herniated disc compressing the spinal cord (which typically have a slower onset and occur in older adults) are less likely. Sudden onset suggests stroke, slow chronic onset tumor, and subacute onset inflammatory disease. The patient's age and gender bring multiple sclerosis (MS) to mind, but her presentation is suggestive of a single lesion in the spinal cord. As the name of the disease indicates, MS is characterized by having more than a single lesion; the lesions of MS are also most often in the brain, rather than the spinal cord, so this is less likely. The patient does not describe her weakness as ascending and her lower extremity DTRs are hyperactive rather than lost, so Guillain-Barré syndrome is less likely. The most likely diagnosis for the patient's current presentation is an inflammatory spinal cord syndrome impairing motor, sensory and autonomic function.

Transverse myelitis is a syndrome of acute or subacute spinal cord dysfunction resulting in paresis, sensory impairment, and autonomic nervous impairment below the level of a lesion.^2^ The tracts affected are spinothalamic (pain, crude touch, and temperature sensation), posterior column medial lemniscus (vibratory, fine touch, and positional sensation), and corticospinal (motor function). Bladder, bowel, and sexual dysfunction are manifestations of autonomic nervous system involvement. The myelitis can be either partial (involving one particular tract or causing asymmetric dysfunction) or total (involving all tracts bilaterally).^2^ Transverse myelitis is the best category for this patient's neurological syndrome, but this categorization is only a starting point; treatment and prognosis depend on the specific cause.

### What is the differential diagnosis for the cause of transverse myelitis?

The causes of transverse myelitis can be broadly classified as parainfectious, paraneoplastic, drug/toxin-induced, systemic autoimmune disorders, and acquired demyelinating diseases such as MS and neuromyelitis optica spectrum disorder (NMOSD).^2^ Transverse myelitis can also lack a distinct cause and is idiopathic in 15–30% of cases.^2^ Spinal epidural abscess mimics transverse myelitis and is an important diagnosis not to miss.^3^

### What is the most likely cause of this patient's transverse myelitis, based on the history and physical examination?

The classic clinical triad of a spinal epidural abscess is focal spinal back pain, fever, and neurologic deficit, but not all patients have this triad.^3^ Most patients have back pain, which is often the first symptom. This patient had no back pain or fever, so a spinal epidural abscess is unlikely.

The infections associated with parainfectious transverse myelitis include *Borrelia burgdorferi*, *Chlamydia psittaci*, mumps virus, cytomegalovirus, coxsackie virus, *Mycobacterium tuberculosis*, *Mycoplasma pneumoniae*, enterovirus 71, hepatitis C virus, *Brucella melitensis*, Epstein-Barr virus, echovirus type 30, *Ascaris suum*, *Toxocara canis*, and *Schistosoma* species.^2^ Case reports of transverse myelitis associated with COVID-19 have appeared.^4^ This patient had no signs or symptoms of these various infections, so parainfectious transverse myelitis is unlikely.

Myelitis can be a manifestation of systemic lupus erythematosus, but this is rare, occurring in only 1–2% of cases.^5^ As indicated in the name of the disease, systemic lupus erythematosus affects multiple organ systems, and patients with myelitis due to lupus typically have manifestations in other organ systems and already carry a diagnosis of lupus, so lupus is unlikely to be the cause of transverse myelitis in this patient.

Sarcoidosis is a granulomatous inflammatory disorder that most often involves the lungs and lymph nodes in young adult female patients, but involves the nervous system alone in 5–15% of cases.^6^ Neurosarcoidosis can cause an intramedullary spinal cord lesion, optic neuritis, ring-enhancing cerebral lesions, a cerebellar nodule or other types of lesions, but each type of lesion is rarely caused by sarcoidosis.^6^ Without other features of sarcoidosis, this diagnosis is unlikely.

Adding the past medical history of episodic blurred vision and headache to the history of the present illness and physical examination narrows the differential diagnosis. It brings MS to the forefront because it represents a lesion separated in space and time from the spinal cord lesion in the current presentation. Lesions in different nervous system locations and at different times are of the essence of MS.

Transverse myelitis can be a manifestation of MS.^2^ An episode of optic neuritis characterized by unilateral painful visual impairment with the gradual recovery of vision over months is a common initial manifestation of MS.^7^ An attack of sensory loss below a spinal cord level due to partial transverse myelitis is another common initial manifestation of MS.^2^^,^^7^ A history of relapsing-remitting attacks of diplopia due to internuclear ophthalmoparesis would strongly suggest MS. A history of relapsing-remitting attacks of dysarthria, facial numbness, vertigo, or ataxia would also suggest MS. This disease is common, with a prevalence of 30–80 per 100,000 in the northern United States and 6–14 per 100,000 in the southern United States.^8^ Onset is almost always in young adulthood (peaking at 30 years of age).^8^ MS is 2–3 times more common in women.^8^ At first impression, it would seem that MS is the most likely cause of this patient's transverse myelitis.

Simultaneous or sequential transverse myelitis and optic neuritis are the classic manifestations of NMOSD.^9^ NMOSD has a low overall prevalence of 0.3–4.4 per 100,000, but the true prevalence is likely significantly higher because up to 40% of cases are misdiagnosed as MS.^10^ NMOSD accounts for 1–2% of demyelinating disease in Caucasians, but 20–48% in Asians.^10^ It typically has onset in young adulthood and is 3–9 times more common in women.^11^ This patient had episodic visual impairment that sometimes lasted for several days. Though associated with a headache, the duration of visual loss was too long to be from a migraine and is more suggestive of ongoing inflammation or optic neuritis. While the patient's optic neuritis was transient and milder than that typically seen in either NMOSD or MS, the optic neuritis of NMOSD is typically worse than that of MS.^12^

The sequential optic neuritis, and transverse myelitis, and demographic features suggest that NMOSD may be the most likely cause of her transverse myelitis, but tests are needed to make a specific diagnosis.

## Diagnostic findings, Part 2

**Fig. 1:**
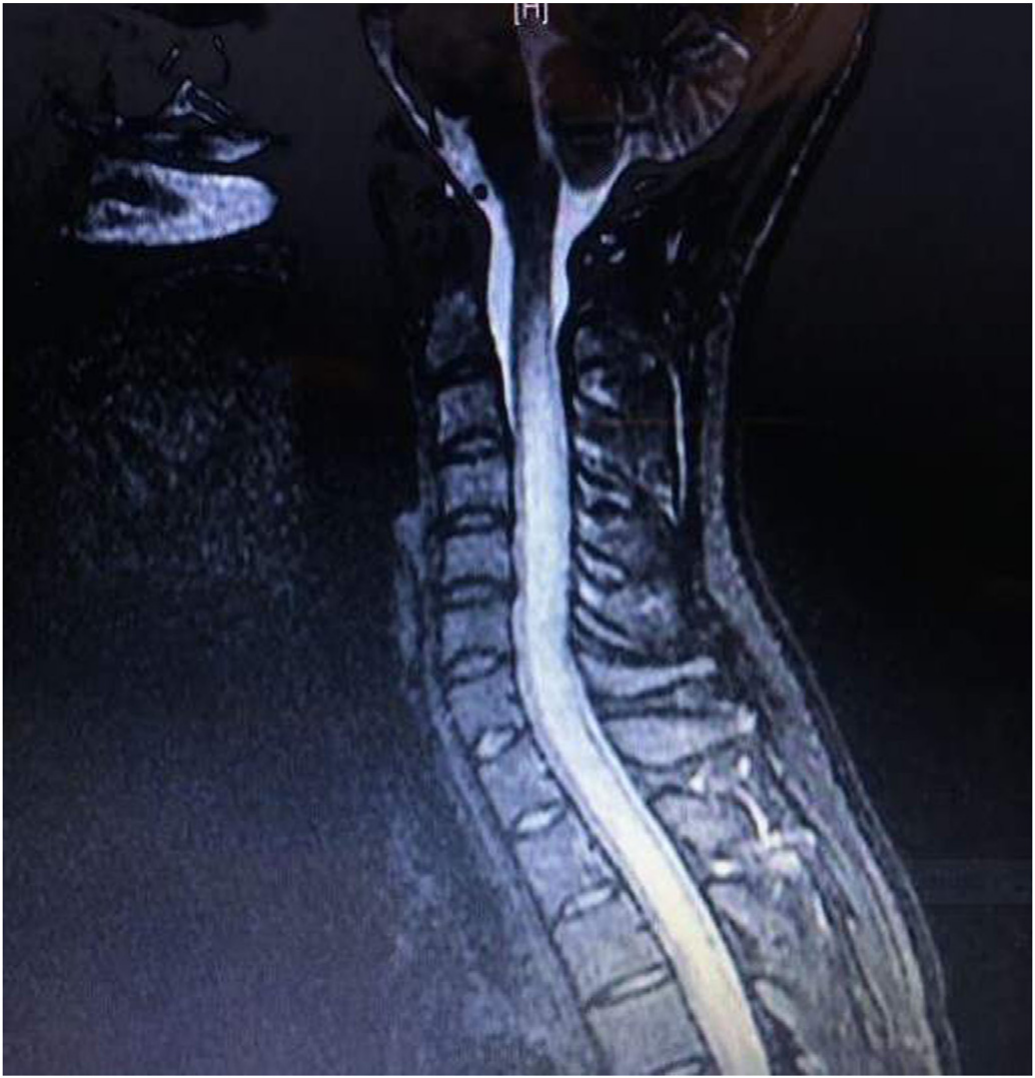
Sagittal T2 weighted Magnetic Resonance Imaging (MRI) of the cervical and upper thoracic spinal cord revealed a contiguous T2 brightness throughout most of the cervical spinal cord and the top half of the thoracic cord (over ten segments), representing a longitudinally extensive lesion/transverse myelitis, a finding characteristic of NMOSD and not typically seen in multiple sclerosis. NMOSD, neuromyelitis optica spectrum disorder.

MRI of the cervical and thoracic spine demonstrates pathological T2 brightness throughout most of the cervical cord and the top half of the thoracic cord (over ten segments), as shown in Fig. 1. A lumbar puncture is performed and the results are displayed in Table 1.

**Table 1:** Laboratory results.

Test	Specimen	Patient's result	Reference range
Protein	CSF	106 mg/dL	20–45 mg/dL
White blood cells	CSF	21 WBCs/uL −97% lymphocytes	0-5 WBCs/uL
Glucose	CSF	55 mg dL	40–70 mg/dL
Oligoclonal bands	CSF	None	None
Anti-aquaporin 4 antibodies	Serum	Positive 1:100	Negative <1:10
Anti-myelin oligodendrocyte antigen antibodies	Serum	Negative	Negative

## Questions/discussion points, Part 2

### The MRI findings best support which diagnosis?

The MRI findings, in this case, are much more likely due to NMOSD than MS.

NMOSD typically causes a continuous spinal cord lesion spanning 3 or more complete vertebral segments, which is termed longitudinally extensive transverse myelitis, and this is most often in the cervical and upper thoracic regions.^13^ In contrast, when MS involves the spinal cord, it typically causes myelitis spanning 1 complete segment or less.^13^

### What diagnosis do the CSF findings favor?

Oligoclonal bands of protein in the CSF, but not in a paired serum sample, are evidence of intrathecal production of antibodies, and their presence supports a diagnosis of MS.^7^ The lack of oligoclonal bands in the CSF, in this case, goes against the diagnosis of MS. The test is performed on concurrently collected CSF and serum specimens, optimally by isoelectric focusing with anti-IgG antibody immunoblotting. A positive evaluation (e.g. three or more antibody bands identified in CSF and not in serum) supports a diagnosis of MS.^7^ Oligoclonal bands are seen in roughly 16% of patients with NMOSD and are typically a transient finding.^14^ Pleocytosis (leukocytes in the CSF) can be a feature of MS, but typically only mild lymphocytic pleocytosis.^7^ Patients with NMOSD typically have mild pleocytosis, with an average of 19 white blood cells/uL.^14^ During an acute episode or relapse, the CSF will show a higher degree of pleocytosis. Patients with MS typically have a mildly elevated level of protein in their CSF, less than 100 mg/dL.^7^ Patients with NMOSD typically have a mildly elevated level of protein level in their CSF, and the level correlates with the length of the spinal cord lesion.^14^ The CSF protein is highest when both optic neuritis and longitudinally extensive transverse myelitis are present, followed by only myelitis present, and lowest when only optic neuritis is present.^14^ A CSF protein level over 100 mg/dL is almost exclusively due to a relapse.^14^ The CSF findings, in this case, favor a diagnosis of NMOSD.

## Diagnostic findings, Part 3

A cell-based assay of the patient's serum assessing for anti-aquaporin 4 antibodies and anti-myelin oligodendrocyte glycoprotein (MOG) antibodies is ordered. Additionally, a titer further assessing anti-aquaporin 4 antibodies is performed. The results can be found in Table 1.

## Questions/discussion points, Part 3

### How diagnostic of NMOSD is finding serum antibodies to aquaporin 4 (AQP4) and how does this relate to the pathogenesis of the disease?

Antibodies of IgG class against AQP4 (AQP4-IgG) can be identified in roughly 75% of NMOSD cases.^15^ A positive cell-based assay for AQP4-IgG has a specificity of 90.6%–100% for the diagnosis of NMOSD.^16^ A cell-based assay has a slightly higher sensitivity (76%) and specificity (99%) when compared to a tissue-based assay (59.0%; 97.0%) and an enzyme-linked immunosorbent assay (65%; 97%).^15^ AQP4 channels are present in the foot processes of astrocytes that are direct contact with the pia mater, capillaries, neurons and in ependymal cells.^15^ The highest concentrations are in the grey and white matter of the spinal cord, ependymal cells lining the ventricles, and in astrocytes lining the optic nerves. Antibody binding to the extracellular domain of the AQP4 receptor results in complement- and cell-mediated injury to the astrocytes, diminished support for surrounding cells such as oligodendrocytes and neurons, breakage in the blood-brain barrier, infiltration by neutrophils, oligodendrocyte damage and demyelination.^11^

AQP4-IgG is mainly IgG 1, a potent activator of the classical complement pathway, which is primarily produced by B cells with the biomarkers CD19, CD27, and CD38.^10^ Some germline B cell clones contain AQP4 B cell receptors that act as antigens to activate T cells against AQP4; additionally, the activated APQ4-specific B cells can act as antigen presenting cells to activate T cells.^10^ Microglial cells can act as antigen-presenting cells to T cells that migrate to cervical lymph nodes and can activate T cells locally within the nervous system parenchyma.^17^ The main activated T cell population seen in NMOSD is debated and may be either TH1 or TH17.^10^ The AQP4 specific T cells can disrupt the blood-brain barrier and facilitate the entry of AQP4-IgG into the central nervous system.^10^ AQP4-IgG can pass through the blood-brain barrier and bind to AQP4, inducing complement-dependent cytotoxicity and antibody-mediated cytotoxicity (by activating natural killer cells) against the AQP4 expressing cells and a pathologic loss of AQP4.^10^ The activation of the complement system expedites the damage to the blood-brain barrier and to astrocyte membranes.^10^ The combination of complement activation and astrocyte damage leads to granulocyte recruitment that can damage oligodendrocytes and lead to a loss of the myelin sheath and axonal damage.^10^ On autopsy, it is common to see complement and NMO-IgG deposition in areas of high AQP4 concentration.^17^

Among patients seronegative for AQP4-IgG, a different antibody against MOG has been identified in roughly 40% of cases.^11^ MOG is a surface component of oligodendrocytes and central nervous system myelin.^11^ While cases of patients with anti-MOG positive status can be grouped under NMOSD, it has been proposed that anti-MOG positive central demyelination disease is an entity pathologically distinct from both MS and NMOSD.^11^^,^^18^

**Fig. 2:**
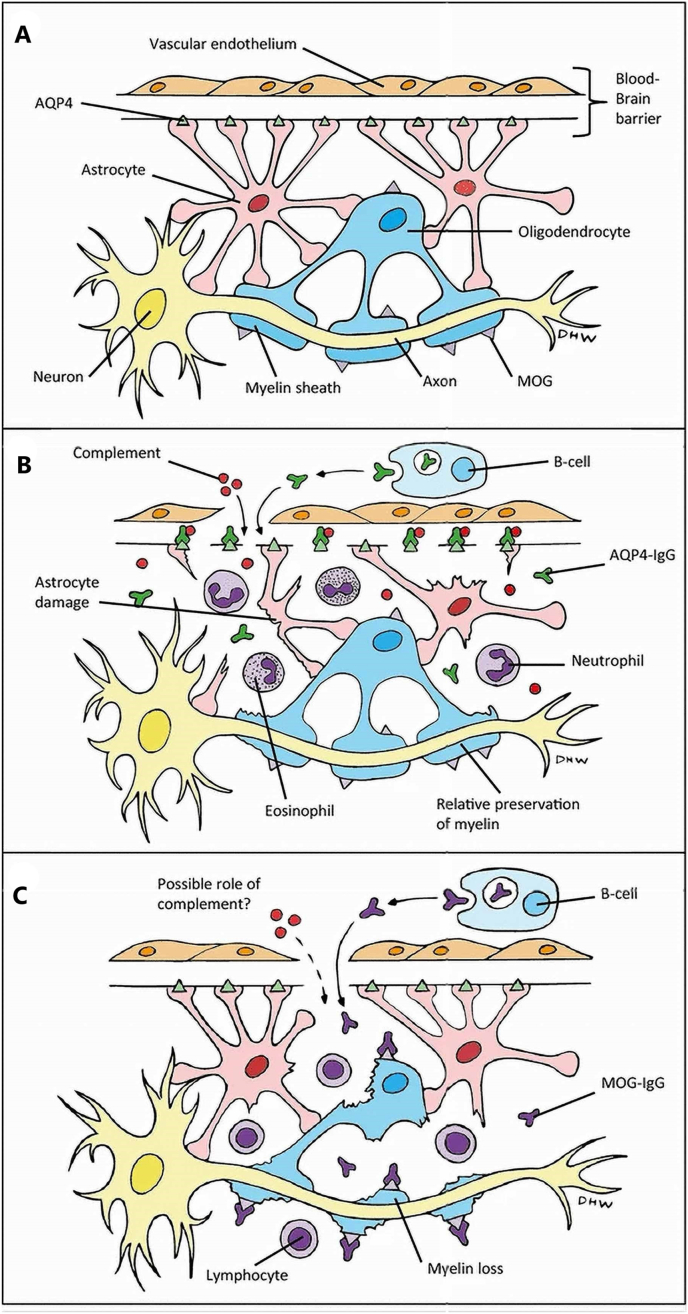
**A**) This diagram depicts the locations that AQP4 and MOG are expressed within the central nervous system (CNS): AQP4 can be found on the foot processes of astrocytes lining the blood-brain barrier while MOG is located on the external layer of CNS myelin and is expressed by oligodendrocytes. (**B**) NMO-IgG is synthesized by systemically circulating B cells. Upon entry of the CNS, NMO-IgG binds AQP4 and activates complement-dependent cytotoxicity that damages the astrocytes and initially spares the myelin. Neutrophils and eosinophils are recruited during the inflammatory process. (**C**) MOG-IgG is also produced from B cells outside the CNS and leads to demyelination. The mechanism is not fully understood. Reprinted by permission from Copyright Clearance Center: Springer Nature: Whittam D, Wilson M, Hamid S et al. What's new in neuromyelitis optica? A short review for the clinical neurologist. J Neurol 2017; 264:2330–44. https://doi.org/10.1007/s00415-017-8445-8. AQP4, aquaporin4; MOG, anti-myelin oligodendrocyte glycoprotein.

A visualization of the pathogenesis of NMOSD can be found in Fig. 2.

### What are the main clinical features of NMOSD?

The main clinical features of NMOSD are painful bilateral optic neuritis that can result in severe vision loss, longitudinally extensive transverse myelitis, acute brainstem syndromes (ipsilateral cranial nerve deficits with contralateral tract deficits), diencephalic lesions presenting as narcolepsy, and area postrema syndrome (lesion in the dorsal medulla presenting as unexplained nausea, vomiting, and/or persistent hiccups).^9^^,^^12^ The main symptoms involve areas of the central nervous system with high concentrations of AQP4.^11^ NMOSD can be either monophasic or polyphasic, with relapsing episodes. Roughly 25% of patients with NMOSD have a coexisting autoimmune disorder such as systemic lupus erythematosus, myasthenia gravis, or Sjogren's disease.^11^

MRI findings of NMOSD include longitudinally extensive transverse myelitis that can extend into the spinal cord medulla, dorsal medullary lesions, and brainstem lesions in periependymal areas.^9^ The optic nerve lesions typically involve over half of the optic nerve and may extend into the optic chiasm; they can be detected on T2 hyperintense MRI or T1 MRI with gadolinium enhancement.^9^

### Why is it critical to distinguish NMOSD from MS?

NMOSD and MS have important differences in treatment, making it critical that patients with NMOSD be appropriately diagnosed. Treatment failure and adverse effects have been reported in patients with NMOSD who were misdiagnosed and initially treated with MS therapies, such as interferon gamma and natalizumab.^10^ The identification of anti-AQP4 antibodies in patients with NMOSD has provided critical insight into the pathogenesis of NMOSD, enabling specific diagnostic tests and treatments.^10^^,^^11^

NMOSD is an autoimmune disease. MS is also an autoimmune disease, but NMOSD is more directly linked to antibodies circulating in the bloodstream. A summary of features differentiating NMOSD and MS can be found in Table 2.

**Table 2:** Differences between neuromyelitis optica spectrum disorder and multiple sclerosis.

	NMOSD	MS
Epidemiology	- 0.3–4.4/100,000 - More common in those of Asian and African descent - 3–9x more common in women	- 30–80/100,000 in the northern US/6–14/100,000 in southern US - 2–3x more common in women
Magnetic resonance imaging		
- Brain	- Dorsal medulla - Periependymal brainstem	- “Dawson fingers” adjacent to lateral ventricle - Inferior temporal lobe - Cerebral cortex
- Optic nerve and chiasm	- Involves ≥ ½ of optic nerve - Favors posterior optic nerve - Affects optic chiasm	- Short, focal lesions
- Spinal cord	- Transverse lesions involving ≥ 3 complete, continuous segments	- Peripheral lesions typically involving ≤1 complete segment
Cerebrospinal fluid		
- Oligoclonal bands	- 16%	- 85%
Serum		
- Anti-aquaporin 4 antibodies	- 75%	- Rare
- Anti-myelin oligodendrocyte glycoprotein antibodies	- Small percent, associated with negative AQP4-IgG status - May represent a different disease process	- Rare

## Diagnostic findings, Part 4

The patient is treated with simultaneous plasmapheresis and steroid therapy. She undergoes five cycles of plasmapheresis administered every other day, and receives one gram of intravenous methylprednisolone per day for five days, followed by a tapering dose, with substantial improvement in her clinical condition beginning at approximately day 10 of treatment.

## Questions/discussion points, Part 4

### What is the treatment and prognosis for patients with NMOSD?

Treatment for acute NMOSD episodes is intravenous glucocorticoid therapy with methylprednisolone. This can be combined with apheresis. Apheresis is a term from Greek for “a taking away”. It is a medical technology taking a patient's blood and passing it through an apparatus that separates out one particular constituent and returns the remainder to the patient's circulation. If the constituent removed is from plasma, it can be termed plasmapheresis. This can also be called therapeutic plasma exchange since the patient's plasma is removed and replaced with an appropriate fluid while returning all the cellular components of the patient's blood. Cellular components such as leukocytes can be removed by apheresis. Since lymphocytes play a role in the pathogenesis of NMOSD, lymphoplasmapheresis is an option.^19^

Apheresis is most beneficial if initiated early in the course of NMOSD, but it can be beneficial even if treatment is delayed.^20^ Therapeutic apheresis also has a role in the management of sickle cell anemia, thrombotic thrombocytopenia, acute and chronic inflammatory demyelinating polyneuropathy, myasthenia gravis, anti-glomerular basement membrane disease, organ transplantation, plasma cell dyscrasias, leukemia, and lymphoma.

Long-term treatment of NMOSD revolves around immunomodulation. New medications for chronic NMOSD include eculizumab, satralizumab, and inebilizumab. Eculizumab is an antibody that binds to the complement component C5 and inhibits the formation of C5b-induced membrane attack complex.^21^ Satralizumab is an antibody that binds interleukin-6 (IL-6) receptors, thereby suppressing inflammation mediated by IL-6 signaling pathways, blocking the effect of IL- 6 released by astrocytes when antibodies bind to the AQP4 channels.^21^ Inebilizumab is an antibody that binds to the CD19 surface antigen of B cells, depleting a wide range of lymphocytes of B cell lineage, including peripheral blood CD20 plasmablasts and plasma cells, including the producers of AQP4 antibodies.^10^^,^^21^ While all three of these drugs have a positive safety profile, they are very expensive and may not be an affordable treatment option for many patients.^21^ Tocilizumab is an IL-6 receptor antagonist antibody alternative to satralizumab.^22^ An alternative antibody treatment with a different target is rituximab, which causes B cell depletion by binding to the CD20 antigen of B cell lymphocytes and to Fc receptors.^23^ These antibody treatments are given intravenously, except for satralizumab (given subcutaneously), and none may be a practical or preferred option for many patients.

After 5 years of disease onset, 50% of untreated NMOSD patients are wheelchair-bound and blind, and 1/3 will have died.^11^ However, treatment with apheresis and monoclonal antibodies can reduce the likelihood of a relapse episode individually or when used together, reducing the possibility of permanent disability and death.^24^ NMOSD has evolved from a misunderstood disorder with almost universally poor outcomes to a disease with specific diagnostic tests and effective treatments different from MS. It is important not to misdiagnose NMOSD as MS.

## Teaching points

• Transverse myelitis is a syndrome of acute or subacute spinal cord inflammation that causes dysfunction resulting in limb muscle weakness, sensory deficits, and autonomic nervous impairment below the level of a lesion.
• The etiology of transverse myelitis can be parainfectious, paraneoplastic, drug/toxin-induced, systemic autoimmune disease-related, or from the demyelinating diseases such as MS or NMOSD.
• NMOSD is a rare group of disorders most common in young women of Asian or African descent.
• NMOSD includes patients with AQP4-IgG and at least one core clinical characteristic (optic neuritis, acute myelitis, area postrema syndrome, acute brainstem syndrome, acute diencephalic clinical syndrome with NMOSD-typical MRI lesions, cerebral syndrome with NMOSD-typical lesions), and with the exclusion of alternative diagnoses; more stringent clinical criteria for diagnosis are required without AQP4-IgG or when serologic testing is unavailable.
• MS is far more common than NMOSD in the United States, and MRI can help differentiate these two conditions.
• Characteristic findings of NMOSD on MRI include a continuous spinal cord lesion spanning at least 3 segments, lesions in the dorsal medulla, lesions in periependymal areas, and extensive optic nerve lesions.
• Common CSF findings of NMOSD include no detection of oligoclonal bands, mild pleocytosis, and mildly elevated protein. Concentration of protein and degree of pleocytosis increase during a relapse episode.
• The presence of serum antibodies to AQP4 is a specific finding that helps make a diagnosis of NMOSD and provides a basis for therapy.
• NMOSD is an antibody-mediated disease that triggers complement activation along with T cell and natural killer cell activity resulting in demyelination and axonal damage in AQP4 dense areas of the nervous system.
• Treatment for acute episodes of NMOSD is intravenous methylprednisolone, which can be combined with apheresis.
• Apheresis can also be used to treat sickle cell anemia, thrombotic thrombocytopenia, acute and chronic inflammatory demyelinating polyneuropathy, myasthenia gravis, anti-glomerular basement membrane disease, organ transplantation, plasma cell dyscrasias, leukemia, and lymphoma.
• Long-term treatment of NMOSD revolves around immunomodulation. Antibody treatments for chronic NMOSD include eculizumab, satralizumab, inebilizumab, tocilizumab, and rituximab.

## Funding

The article processing fee for this article was funded by an Open Access Award given by the Society of ‘67, which supports the mission of the Association of Pathology Chairs to produce the next generation of outstanding investigators and educational scholars in the field of pathology. This award helps to promote the publication of high-quality original scholarship in *Academic Pathology* by authors at an early stage of academic development.

## Declaration of competing interest

The authors of Educational Case: Neuromyelitis Optica, did not receive any funding to write the article. This research received no specific grant from any funding agency in the public, commercial, or not-for-profit sectors.
